# Preliminary outcomes of the combination of demineralized bone matrix and platelet Rich plasma in the treatment of long bone non-unions

**DOI:** 10.1186/s12891-021-04840-2

**Published:** 2021-11-15

**Authors:** Wei Nie, Zhaojun Wang, Jiaqing Cao, Wei Wang, Yanjie Guo, Chi Zhang, Weitao Jia, Xiaolin Li

**Affiliations:** 1 Department of Orthopedic Surgery, Lianyungang 2nd People’s Hospital, 41 Hailian Road, Haizhou District, Lianyungang, Jiangsu Province China; 2 Department of Orthopedic Surgery, Shanghai 6th People’s Hospital, Shanghai Jiao Tong University, 600 Yishan Road, Xuhui District, Shanghai, China

**Keywords:** Demineralized bone matrix (DBM), Platelet rich plasma (PRP), Bone non-unions, Surgical treatment

## Abstract

**Background:**

A variety of bone graft substitutes have been introduced into the treatment of bone non-unions. However, clinical outcomes from current evidences are various and conflicting. This study aimed to present the preliminary outcomes of a treatment protocol in which the combination of demineralized bone matrix (DBM) and platelet rich plasma (PRP) was used as a bone graft substitute for long bone non-unions.

**Methods:**

Data of this retrospective study were reviewed and collected from a consecutive case series involving 43 patients who presented with a long bone non-union and were treated in our department from October 2018 to May 2019. The combination of DMB and PRP was applied as a bone defect filler in 16 patients, whilst the other 27 patients were treated with iliac bone autografting. Patients’ demographics, postoperative complications and the result of bone union were compared and evaluated.

**Results:**

The demographic data between the two groups were comparable. No significant difference was found with regard to the incidence of postoperative complications. No graft rejection, heterotopic ossification or other complications were noted. The distribution of bony healing time was rather scattered but did not differ significantly between the groups (7.533 ± 3.357 months vs. 6.625 ± 2.516 months; *P*=0.341). Union was identified radiographically in 15 of 16 patients in the DBM+PRP group and in 24 of 27 patients in autograft group.

**Conclusions:**

The present study identified that low incidence of postoperative complications and satisfactory bony healing rate could be achieved in the treatment of long bone non-unions augmented with the combination of DBM and PRP. Although these findings might indicate the promising future of this treatment protocol, larger and higher quality studies should also be executed to assess its routine use.

**Supplementary Information:**

The online version contains supplementary material available at 10.1186/s12891-021-04840-2.

## Background

With a prevalence estimated to be 5 %-10 % in long bones [1], the non-union is a disabling disease characterized by the cessation of bone regeneration and the failure of fracture healing. Treatment of non-unions is highly individualized, long lasting and burdensome, and normally requires a dramatic and effective utilization of resources [2, 3]. Autografting is universally recognized as the mainstay of therapy for non-unions since the autogenous bone is equipped with the essential elements required in bone regeneration in term of osteoconductivity, osteoinductivity and osteogenesis. Despite this, notable concerns about autografts are the limited supply and donor site morbidities, which restricted the application of autogenous bone and aroused practitioners’ desire for seeking for other optimal solutions [4]. Several strategies have been introduced, such as bone marrow aspirate, allografts, composite artificial bone and biological factors. Although attractive, these novel substitutes have yielded inconsistent results when they were used in clinical practices, either combined or alone [5–7].

Both demineralized bone matrix (DBM) and platelet rich plasma (PRP) are current available alternatives for autografting and were used as important therapeutic options in orthopedic surgeries. DBM is decalcified allogeneic bone tissue which still a variety of osteoinductive proteins, mainly including bone morphogenetic proteins (BMP) -2, -4, and -7 [5, 8]. However, when it was used as a bone defect filler in the treatment of non-unions, the results were not so encouraging as the high incidences of complications and low healing rate (approximately 65 % – 84 %) [9, 10]. PRP is versatile autologous blood product with high activated platelet concentration and considerable amounts of growth factors that are able to enhance the recruitment and proliferation of tenocytes, stem cells, and endothelial cells. [11, 12]. It has been shown to be osteopromotive rather than osteoinductive for bone regeneration. Besides, it is reported that PRP cannot produce the desired stimulatory response and provides little benefit in the treatment of non-unions with bone defects when used independently [5, 7, 13].

Theoretically, the application of DBM and PRP together might reinforce the healing of bone non-unions than when they were used independently [14]. The characteristic of DBM allows it can be applied in reconstruction of bone defects and could be mixed with various other materials, such as normal saline, antibiotics solution, autograft, allograft, bone marrow aspirate, whole blood or platelet concentrate [6]. PRP could significantly reinforce the biologic activity of DBM when used as an adjuvant without thrombin activation [14]. Moreover, it can induce active responses in promoting the repair of the soft tissue, thus decreasing the soft tissue-related complications and optimizing the biological environment for bone regeneration. By now, only few animal studies have evaluated the efficacy of the combination of DBM and PRP as a bone substitute and even less articles reported clinical outcomes when it used in the treatment of bone non-unions [15]. We therefore initiated this treatment protocol in which the combination of DBM and PRP was used as a bone graft material to evaluate the outcomes of this combination in in the treatment of long bone non-unions.

## Methods

### Patients Demographics

This retrospective study was approved by the Ethics Committee of Lianyungang 2nd People’s Hospital (2018-037) in accordance with the standards of the Declaration of Helsinki. Data was reviewed from patients with bone non-unions who were treated surgically in our hospital from October 2018 to May 2019. Patient consents were obtained accordingly.

Bony non-union was defined as a fracture that not consolidate for a minimum of 9 months without signs of healing throughout the past 3 months [16], and was confirmed on plain radiographs or by computed tomography (CT) or both. Patients were assessed by clinical symptoms (e.g., deformity, abnormal activities, infection), radiological appearances (e.g., x-ray, CT, PET-CT) and laboratory targets (e.g., white blood cell count, erythrocyte sedimentation rate, C-reactive protein) to identify if a bone infection or an osteonosus exists. Patients with pathological fractures, osteonosus, cancers, immune disorders, thrombocytopenia, platelet dysfunction, large bone defects exceeding 5 cm of diameter, infectious non-unions, and hypertrophic non-unions without any previous therapeutic intervention (e.g., dynamization, exchange nailing) were excluded.

A total of 16 patients with mean age of 41.2 years (ranging from 28 years to 61 years) were treated according to our surgical protocol, whereas the other 27 patients (32, 22-67 years) treated by autografting were included as a comparison group. Preoperative and postoperative data was collected and identified from their past medical records. Causes of injury, localizations of non-union site, types of non-unions, previous surgical interventions and other details of patient demographic data were presented in Table 1.

**Table 1:** Patients Demographics

	DBM+PRP group (n = 16)	Autograft group (n=27)	Statistics	*P* Value
Sex (male/female), n	7/9	15/12	χ^2^ = 0.560	0.454^a^
Age, (years) median, range	39.5,28-61	32, 27-67	*Z*=-0.972	0.331^b^
Affected side			χ^2^ = 0.078	0.78^a^
Right	9	14		
Left	7	13		
Causes of injury			3.918	0.412^c^
Traffic accident	6	14		
Fall	4	9		
Trip	3	3		
Sport injury	2	1		
Machinery accident	1			
Previous Open fracture	5	9	χ^2^ = 0.020	0.888^a^
Number of previous surgeries			3.764	0.226^c^
1	10	19		
2	5	5		
3		3		
4	1			
Location of nonunion site			2.492	0.727^c^
Clavicular	2	2		
Humeral	2	5		
Radial	1			
Femoral	4	9		
Tibial	7	11		
Types of non-union (Oligotrophic/ Atrophic)	13/3	20/7	χ^2^ = 0.027	0.869^d^
Hardware of the last surgery			3.859	0.139^c^
Intramedullary nail	2	7		
Plate	14	16		
External fixation		4		
Chronic disease				
Hypertension	4	6	χ^2^ = 0.000	1.000^d^
Diabetes	3	3	χ^2^ = 0.059	0.808^d^
Duration of non-union (months), mean ± SD	15.94±3.75	16.11±4.24	*t*=-0.135	0.893^e^
Follow-up period (months), mean ± SD	15.28±2.58	16.30±3.23	*t*=-0.970	0.338^e^

Note. ^a^: Pearson’s chi-square test; ^b^: Mann-Whitney U test; ^c^: Fisher’s exact test; ^d^: continuity correction chi-square test; ^e^: independent-samples T test

### Surgical procedures

**Fig. 1:**
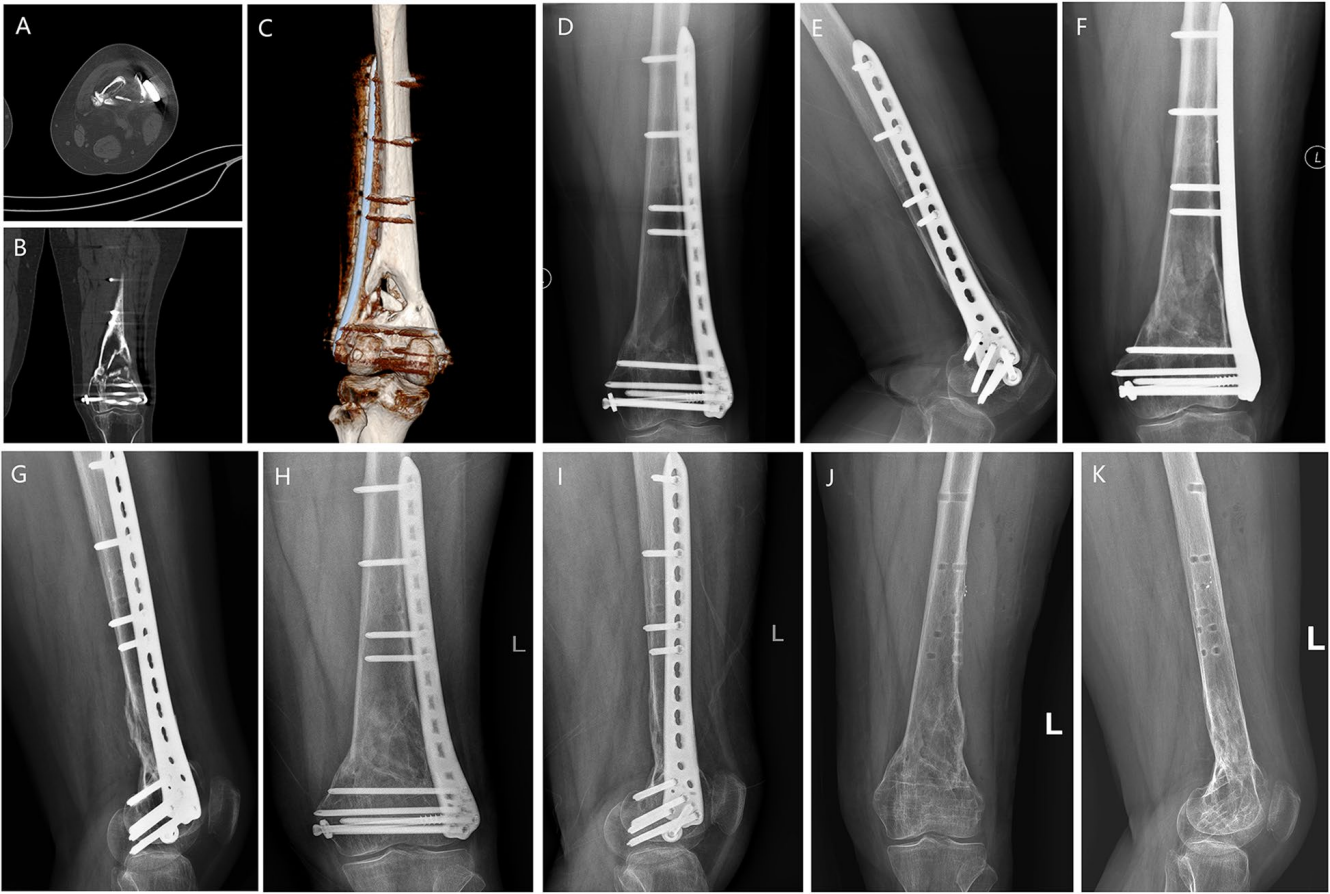
The CT scans (**A**, **B**, **C**) and the preoperative X-ray films (**D**, **E**) identified the non-union of femoral supracondyle. The Judet decortication technique was used to expose the non-union sites, and the paste comprised of DMB, PRP and allograft was implanted to fill the bone defect, covered with allogenous cortical bone around. Postoperative anteroposterior (**F**) and lateral (**G**) position films of X-ray were taken 3 days after surgery. The follow-up radiographic image showed bony union was achieved at 6 months (**H**, **I**). The hardware was removed 16 months postoperatively (**J**, **K**)

All surgical procedures were performed by the same surgical team and followed a specific operation-protocol. After anesthesia, 50 ml of autogenous blood was drawn from peripheral vein and was isolated by two-step centrifugation for the preparation of PRP (5ml). During operation, the periosteum and soft tissues surrounding the non-union site was carefully protected to avoid devascularization when exposing. Radically debridement of intervening scar tissue between the non-union site and re-open of medullary canals were performed to allow rapid neovascularization and migration of osteogenic cells. For the DM+PRP group, the bone defect was filled with the paste mixed by DBM putty (Allomatrix, Wright Medical Technology, Inc. Memphis, TN, USA) and PRP at a ratio of 5:1. In patients with big size of bone defects, allogeneic bone was used for volume augmentation if necessary, and the amount was determined by the size of the defect and the experience of surgeons. For the autograft group, autologous iliac bone was used as a bone defect filler. The initial hardware was not removed routinely unless they were loosened or there were requirements for debridement, fixation revision or deformity correction (Fig. 1). An additional plate would be implanted depending on the demand of mechanical stability. After the placement of a suction drain, standard wound closure and pressure dressing were performed.

### Postoperative management and follow-up

All patients were treated with perioperative antibiotic prophylaxis and were informed to quit smoking. Low-molecular weight heparin was given for 2 weeks for patients undergoing the lower extremity surgery. Anteroposterior and lateral position X-ray films were taken within 3 days postoperatively. To prevent the potential wound problems such as seroma, incision exudation and infections, the suction drain was normally kept for 2 days and was removed if the daily drainage volume was less than 30ml/24 h. Otherwise, the suction drain would be persisted. However, this practice has been changed in 1 patient, his drain was removed on postoperative day 7 even though the last daily drainage volume was more than 45 ml. Passive and active range-of-motion without weight-bearing were encouraged for early rehabilitation within the first 4 weeks, and then weight-bearing and strengthening exercises were recommended depending on radiological findings. Patients were followed up at monthly intervals postoperatively for radiographic evaluation of bony union, which was defined as the presence of bridging callus formation on at least 3 out of 4 cortices in two different planes and were evaluated by a senior doctor and two radiologists who were independent from this study.

### Outcome measures and statistics

Outcomes including the drainage time, postoperative complications and the time of bony union were reviewed from medical records. The prolonged drainage (beyond 2 days), incision exudation, delayed wound healing or long-term disunion, superficial or deep infections, and graft rejection were defined as postoperative complications. Functional recovery was not assessed because there is lack of a unified criterion when the affected limbs and localizations of non-union site were different between patients.

Categorical data are presented as frequencies and the associations were determined using the χ^2^ test or Fischer’s exact test. Continuous variables are presented as mean or median (range) and were compared using Mann-Whitney U test or two-sample t-test. Statistical analysis was preformed using SPSS 22.0 (Chicago, IL, USA).

## Results

The bone defect sizes varied between patients and allogeneic bone was added for augmentation in 5 cases. No significant differences were observed between the demographic characteristics of the two groups regarding demographics, locations of non-union site, duration of non-unions and constituent ratio of non-union types. In the DBM+PRP group, there were 6 of 16 patients (37.5 %) whose postoperative drainage persisted beyond 48 h. Only in 1 patient the drainage was kept for 7 days and was removed even though there was still a daily drainage quantity more than 45 ml. But no positive symptoms or laboratory findings indicating a superficial or deep infection were found. Incision exudation was observed in 2 patients. Delayed incision healing (exceed 2 weeks) was observed in 2 patients, one of which was the same patients with 7-day postoperative drainage and incision exudation. Subcutaneous hematoma was detected in 1 patient after the removal of the suction drain and was cured with percutaneous aspiration and pressure dressing. In the autograft group, there were 11 of 27 patients (40.7 %) who demonstrated postoperative drainage exceeding 2 days but less than 5 days. Delayed incision healing (exceed 2 weeks) was observed in 4 patients, and incision exudation was also detected in 3 of them. Donor site morbidity happened in 3 patients, including subcutaneous hematoma and fat liquefaction. Of these 3 patients, 1 patient developed superficial infection. They were finally healed after a due course of conservative treatment consisted of intravenous antibiotics, local wound care and compressive dressings. These details were summarized in Table 2 and additional file 1. There was no graft rejection, heterotopic ossification or any other complications related to surgeries had been noted, and no one needed an additional surgical intervention.

**Table 2:** Surgical protocol and postoperative outcomes

	DBM + PRP group	Autograft group	Statistics	*P* Value
Types of operations			χ^2^ = 0.148	0.700^a^
Bone grafting	5	10		
Bone grafting + additional plate	11	17		
Bone grafting materials				
DMB + PRP	11			
DMB + PRP + allograft	5			
Autologous iliac bone		27		
Immediate postoperative complications				
Prolonged drainage	6	11	χ^2^ = 0.044	0.834^a^
Exudation	2	3	χ^2^ = 0.000	1.000 ^b^
Delayed incision healing	2	4	χ^2^ = 0.000	1.000^b^
Subcutaneous hematoma	1			0.372^c^
Donor site complications		3		0.282^c^
Bony healing time (months), mean ± SD	7.533 ± 3.357	6.625 ± 2.516	*t*=0.964	0.341^d^
Recurrence of bone non-union	1	3	χ^2^ = 0.000	1.000^b^

Note. ^a^: Pearson’s chi-square test; ^b^: continuity correction chi-square test; ^c^: Fisher’s exact test; ^d^: independent-samples T test

**Fig. 2:**
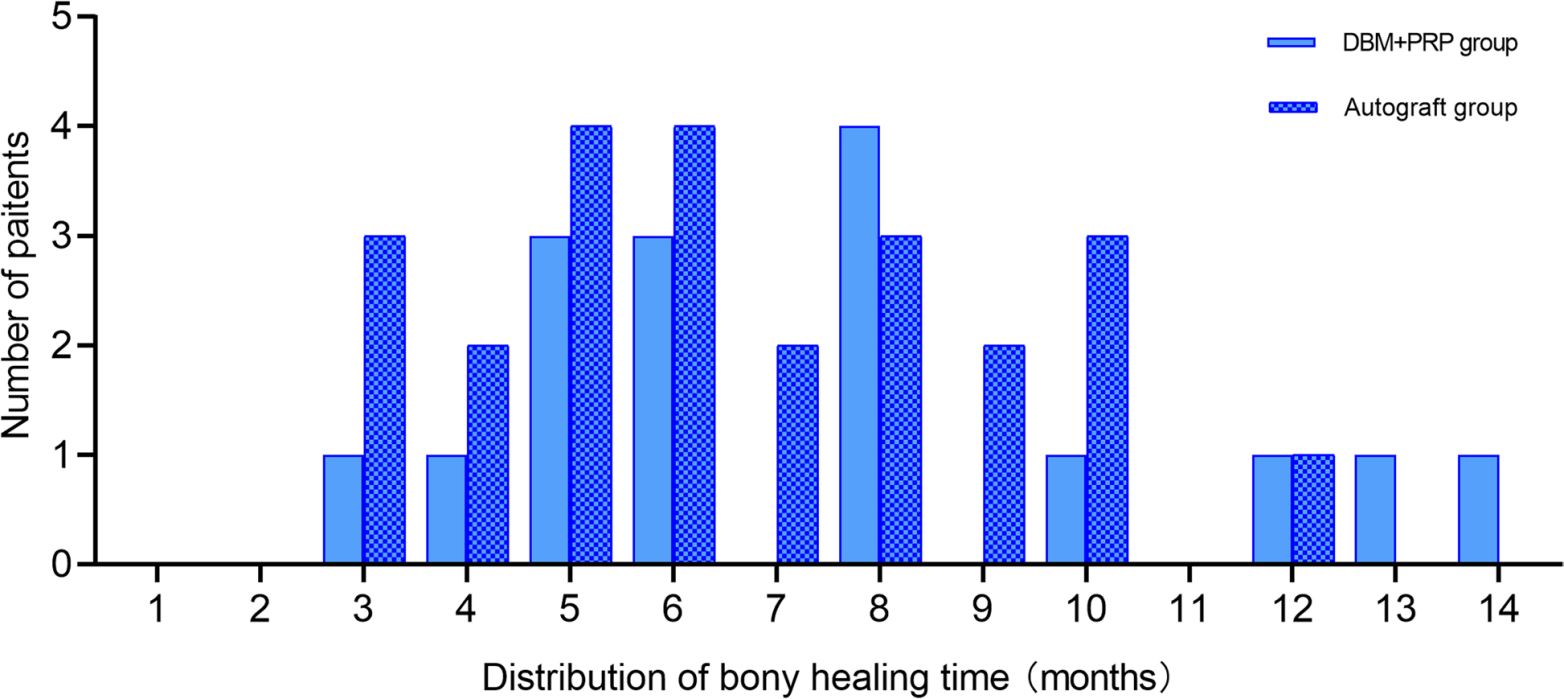
Column chart outlining the distribution of bony healing time in the two groups (7.533 ± 3.357 months vs. 6.625 ± 2.516 months, *t*=0.964d, *P*=0.341)

Bony healing time (7.533 ± 3.357 months vs. 6.625 ± 2.516 months) and the rate of non-union (6.25 % vs. 11.1 %, *P*=1.000) did not significantly differ between the groups (Table 2). In the DMB+PRP group, the formation of bony callus appeared on 9 of the 16 patients’ radiographs within 4 months after the index surgery. Whilst, obvious resorption at the grafting area was also observed in many patients, mainly within 6 months postoperatively. Fifteen patients achieved bony union in radiographical evaluations. However, the distribution of bony healing time was rather scattered, ranging from 3 months to 14 months (mean time 7.533 months) (Fig. 2). A persistent non-union was observed in 1 patient, in whose X-ray showed the appearance of screw loosening, sclerosis and atrophy at fracture sites and no signs of callus formation at his last visit (16 months postoperatively). In the autograft group, the bony healing time ranged from 3 months to 12 months (mean 6.625 months). There was a total of 3 of 27 (11.1 %) patients who failed to achieve bony union and required revision surgeries.

## Discussion

The present study demonstrated promising preliminary results in the surgical management of uninfected long bone non-unions with the combination of DBM and PRP used as a bone graft substitute. Initially, this treatment protocol was mainly applied as a salvage for patients who were unwilling to accept an additional harvesting surgery or who had limited availability of autogenous bone due to the history of previous harvesting surgeries.

Ziran and colleges identified significant incidences of prolonged drainage (51 %), postoperative complications and failure of healing (46 %) in patients with non-unions treated with DBM [9]. However, in view of too many patients with a history of previous infection in their study, the authors inferred that the inadequately treated bone infection might be a main contributor to the occurrences of postoperative complications and the failure of bony healing. They also surmised that a compromised tissue bed might be another possible reason for the development of prolonged drainage and postoperative infections [9]. Encouraging results were found in Hierholzer’s study which retrospectively analyzed 78 patients with non-unions of humeral shaft fractures. The time to union and healing rate in the DBM-augmentation group was comparable to those of the autograft group. The authors also emphasized that the rigid fixation and the protection to local biology of soft tissue played an important role in the success of the treatment [17].

Based upon the experiences from these studies, multiple active measures were taken in our clinical practice, including comprehensive examinations and perioperative antibiotic coverage, careful and radical surgical debridement, minimal injury to the surrounding soft tissues, adequate postoperative drainage, and rigid fixation of the non-union sites, all of which were of great benefits for preventing potential complications and facilitating bone regeneration. Concerning the poor physical stability of the DBM, a crucial principle in our operation was the complete coverage to bone graft materials with periosteum or other surrounding soft tissues so that it could provide a protection to the graft materials and enhance the biological environment for bone regeneration. Benefit from these measures, satisfactory clinical outcomes were observed in this series and the rate of prolonged drainage and failure of bony healing were relatively lower when compared with Ziran’s study [9]. In this series, the combination of DBM and PRP could be regard as a safe and effective bone graft substitute since the incidences of local or systemic complications were relatively low, and bony union was achieved in 15 out of 16 patients at the end of follow-up. Besides, comparing with autogenous or allogenous bone, the combination of DBM and PRP had the advantage in bone defect filling as it can be made into various shapes and different sizes according to the shapes of bone defects. In addition, it could be concluded that the surgical time, as well as multiple associated complications, would be reduced as there is no need for an additional procedure of an autogenous bone harvesting.

The poor condition of soft tissue is considered as a risk factor related to the postoperative wound complications [18]. A finding of the present study is that we noticed most of patients with prolonged drainage duration and incision-related complications have a previous history of open fracture which could result in the poor quality of local soft tissue. By using the Fisher’s exact test, we found that the development of a prolonged drainage and delayed incision healing were correlated with a history of previous open fractures, implying that the type of bone graft materials should not be regarded as a significant independent risk factor to the incidence of postoperative complications. These results were tabulated in additional file 2.

Still another important observation was that an obvious radiolucent area, without bone proliferation or even with bone resorption, appeared on radiographs in many patients. Only some tiny radiopaque bone fragments were visible in the graft area, which were believed to be caused by the surgical debridement and decalcified bone debris of the DBM. That might mislead surgeons into having a suspicion of the recurrence of bone non-unions. The exact mechanism of this phenomenon is difficult to know. It was probably caused by the excellent absorbability of DBM and the slow process of bone proliferation at the non-union site.

As surmised previously, we found no statistical difference between the two groups in terms of bony healing time. However, the bony healing time was various between patients and excessed 9 months in several patients. This phenomenon might be due to the differences in anatomical location of non-unions. Since we had no reference data about the time necessary for bony healing in this treatment protocol, the observation time were mainly determined by the radiographical appearance in which there were signs of progressive bone formation or bony bridge in 3 consecutive months.

Although satisfactory outcomes were obtained in this series, several limitations were identified but difficult to avoided. The constitute ratio of non-union types showed no significant difference between the two groups. However, every non-union has its unique characteristics. Some clinical variables, including the size of bone defect, the sites and types of non-unions and the kinds of previous hardware, were not standardized between patients. These factors might influence the biologic and biomechanical conditions that require a tailored treatment strategy, including correction of deformation, an additional implant for rigid stabilization and augmentation with other additional osteoinductive agents such as allograft bone. Due to the small simple size and the variety of the explanatory variables, only chi-square test and t test were used to analyze the relationships between them, and we could not perform further correlation test or regression analysis.

## Conclusions

Our experience and preliminary clinical data based on this study suggest that, if properly administered, the combination of DBM and PRP could serve as a safe bone graft substitute in clinical practice for non-union, especially for patients with limited availability of autogenous bone or patients with contraindication for an additional harvesting surgery. Due to the limitations of the present study, further rigorous researches, randomized controlled trials or otherwise, are still indicated for the investigation on its routine use.

## Supplementary Information

**Additional file 1.**

**Additional file 2.**

## Data Availability

The datasets used and/or analyzed during the current study are available from the corresponding author on reasonable request.

## Abbreviations

DBM: demineralized bone matrix
PRP: platelet rich plasma
CT: computed tomography
PET: positron emission tomography
