# Supplementary material for: Preliminary outcomes of the combination of demineralized bone matrix and platelet Rich plasma in the treatment of long bone non-unions
Source: BMC Musculoskelet Disord. 2021 Nov 15;22:951. doi: 10.1186/s12891-021-04840-2 (PMC8594103; doi:10.1186/s12891-021-04840-2)
Supplement: Supplementary file 1 — Additional file 1. [file 12891_2021_4840_MOESM1_ESM.docx]

| No. of patients | Sex（Male/Female） | Age (range) | Affected side | Causes of injury | Previous Open fracture | Number of previous surgeries | Location of nonunion site | Types of  non-union | Initial hardware | Chronic disease | Duration of non-union | Follow-up period |
| --- | --- | --- | --- | --- | --- | --- | --- | --- | --- | --- | --- | --- |
| 1 | F | 40's-50's | L | Traffic accident |  | 2 | Femoral supracondyle | Atrophic | Plate | Hypertension | 18 | 16 |
| 2 | F | 40's-50's | L | Traffic accident |  | 1 | Tibial proximal segment | Oligotrophic | Plate |  | 24 | 14 |
| 3 | M | 40's-50's | R | Fall | Y | 4 | Tibial mid-shaft | Oligotrophic | Plate |  | 16 | 18 |
| 4 | F | 60's-70's | L | machinery accidents |  | 1 | radial mid-shaft | Oligotrophic | Plate | Hypertension, diabetes | 18 | 15 |
| 5 | M | 30's-40's | R | Fall |  | 2 | Femoral mid-shaft | Oligotrophic | Plate |  | 16 | 14 |
| 6 | F | 30's-40's | R | Traffic accident | Y | 2 | Tibial mid-shaft | Atrophic | Plate |  | 9 | 18 |
| 7 | M | 30's-40's | L | Trip |  | 1 | Clavicular mid-shaft | Oligotrophic | Plate |  | 18 | 12 |
| 8 | F | 20's-30's | L | Sport injury |  | 1 | Humeral mid-shaft | Oligotrophic | Intramedullary nail |  | 12 | 15 |
| 9 | F | 30's-40's | R | Traffic accident | Y | 2 | Tibial mid-shaft | Atrophic | Plate |  | 20 | 12 |
| 10 | F | 50's-60's | R | Fall |  | 1 | Femoral mid-shaft | Oligotrophic | Plate | Hypertension, diabetes | 14 | 18 |
| 11 | M | 30's-40's | L | Trip |  | 1 | Clavicular mid-shaft | Oligotrophic | Plate |  | 15 | 12 |
| 12 | M | 30's-40's | R | Fall | Y | 1 | Femoral supracondyle | Oligotrophic | Plate |  | 15 | 16 |
| 13 | M | 30's-40's | L | Traffic accident |  | 1 | Tibial mid-shaft | Oligotrophic | Intramedullary nail |  | 12 | 18 |
| 14 | F | 50's-60's | R | trip |  | 1 | Proximal humerus | Oligotrophic | Plate | Diabetes | 20 | 12 |
| 15 | M | 40's-50's | R | Traffic accident | Y | 2 | Tibial mid-shaft | Oligotrophic | Plate |  | 12 | 20 |
| 16 | F | 50's-60's | R | Sport injury |  | 1 | Tibial distal segment | Oligotrophic | Plate | Hypertension | 16 | 16 |

Table 1. Demographic characteristics of patients in DMB+PRP group.

Table 2. Clinical outcomes of patients in DBM+PRP group.

| No. of patients | Sex  （Male/Female） | Age (year) | Surgical methods | Bone graft materials | Persistence of postoperative drainage | Incision-related complications | Incision healing time | Bony healing time（months） |
| --- | --- | --- | --- | --- | --- | --- | --- | --- |
| 1 | F | 40's-50's | Bone grafting | DMB + PRP + allograft | 2 | N | 2 weeks | 6 |
| 2 | F | 40's-50's | Bone grafting + additional plate | DMB + PRP | 3 | N | 2 weeks | 12 |
| 3 | M | 40's-50's | Bone grafting + additional plate | DMB + PRP | 4 | N | 2 weeks | 10 |
| 4 | F | 60's-70's | Bone grafting | DMB + PRP +allograft | 2 | N | 2 weeks | 6 |
| 5 | M | 30's-40's | Bone grafting + additional plate | DMB+PRP | 2 | N | 2 weeks | 5 |
| 6 | F | 30's-40's | Bone grafting + additional plate | DMB + PRP + allograft | 4 | N | 4 weeks | 13 |
| 7 | M | 30's-40's | Bone grafting | DBM + PRP | 2 | N | 10 days | 4 |
| 8 | F | 20's-30's | Bone grafting + additional plate | DMB + PRP | 2 | N | 2 weeks | 5 |
| 9 | F | 30's-40's | Bone grafting + additional plate | DMB + PRP | 3 | N | 2 weeks | 8 |
| 10 | F | 50's-60's | Bone grafting + additional plate | DMB + PRP | 2 | N | 2 weeks | 8 |
| 11 | M | 30's-40's | Bone grafting | DBM + PRP | 2 | N | 10 days | 3 |
| 12 | M | 30's-40's | Bone grafting | DMB + PRP + allograft | 2 | N | 2 weeks | Non-union |
| 13 | M | 30's-40's | Bone grafting + additional plate | DMB + PRP | 2 | Subcutaneous hematoma | 2 weeks | 6 |
| 14 | F | 50's-60's | Bone grafting + additional plate | DMB + PRP | 2 | N | 12days | 5 |
| 15 | M | 40's-50's | Bone grafting + additional plate | DMB + PRP | 7 | Incision exudation | 3 weeks | 14 |
| 16 | F | 50's-60's | Bone grafting + additional plate | DMB + PRP + allograft | 3 | N | 2 weeks | 8 |
