# Supplementary material for: Preliminary outcomes of the combination of demineralized bone matrix and platelet Rich plasma in the treatment of long bone non-unions
Source: BMC Musculoskelet Disord. 2021 Nov 15;22:951. doi: 10.1186/s12891-021-04840-2 (PMC8594103; doi:10.1186/s12891-021-04840-2)
Supplement: Supplementary file 2 — Additional file 2. [file 12891_2021_4840_MOESM2_ESM.docx]

|  |  | Previous open fracture | | Statistic | P value |
| --- | --- | --- | --- | --- | --- |
|  |  | Yes | No |  |  |
| Prolonged drainage | Yes | 10 | 7 | χ^2^=8.833 | 0.003^a^ |
|  | No | 4 | 22 |  |  |
| Delayed incision healing | Yes | 6 | 0 |  | 0.000^b^ |
|  | No | 7 | 30 |  |  |

Table 1. Wound problems in patients with/without a previous history of open fracture.

Note. a: Pearson’s chi-square test; b: Fisher’s exact test.
